# SspA up-regulates gene expression of the LEE pathogenicity island by decreasing H-NS levels in enterohemorrhagic *Escherichia coli*

**DOI:** 10.1186/1471-2180-12-231

**Published:** 2012-10-11

**Authors:** Anne-Marie Hansen, Ding Jun Jin

**Affiliations:** 1 Transcription Control Section, Gene Regulation and Chromosome Biology Laboratory, Frederick National Laboratory for Cancer Research, National Cancer Institute, National Institutes of Health, Frederick, MD 21702, USA; 2 Present Address: Department of Microbiology and Immunology, University of Maryland School of Medicine, Baltimore, MD, 21201, USA

**Keywords:** Enterohemorrhagic *Escherichia coli*, LEE, SspA, H-NS

## Abstract

**Background:**

Enterohemorrhagic *Escherichia coli* (EHEC) colonizes the intestinal epithelium and causes attaching and effacing (A/E) lesions. Expression of virulence genes, particularly those from the locus of the enterocyte effacement (LEE) pathogenicity island is required for the formation of a type three secretion system, which induces A/E lesion formation. Like other horizontally acquired genetic elements, expression of the LEE is negatively regulated by H-NS. In the non-pathogenic *Escherichia coli* K-12 strain the stringent starvation protein A (SspA) inhibits accumulation of H-NS, and thereby allows de-repression of the H-NS regulon during the stationary phase of growth. However, the effect of SspA on the expression of H-NS-controlled virulence genes in EHEC is unknown.

**Results:**

Here we assess the effect of SspA on virulence gene expression in EHEC. We show that transcription of virulence genes including those of the LEE is decreased in an *sspA* mutant, rendering the mutant strain defective in forming A/E lesions. A surface exposed pocket of SspA is functionally important for the regulation of the LEE and for the A/E phenotype. Increased expression of *ler* alleviates LEE expression in an *sspA* mutant, suggesting that the level of Ler in the mutant is insufficient to counteract H-NS-mediated repression. We demonstrate that the H-NS level is two-fold higher in an *sspA* mutant compared to wild type, and that the defects of the *sspA* mutant are suppressed by an *hns* null mutation, indicating that *hns* is epistatic to *sspA* in regulating H-NS repressed virulence genes.

**Conclusions:**

SspA positively regulates the expression of EHEC virulence factors by restricting the intracellular level of H-NS. Since SspA is conserved in many bacterial pathogens containing horizontally acquired pathogenicity islands controlled by H-NS, our study suggests a common mechanism whereby SspA potentially regulates the expression of virulence genes in these pathogens.

## Background

Enterohemorrhagic *Escherichia coli* (EHEC) O157:H7 is an emerging food- and waterborne- enteric pathogen causing diarrhea, hemorrhagic colitis and the potentially fatal complication hemolytic uremic syndrome in humans [1,2]. EHEC colonization of enterocytes of the large bowel is characterized by an intestinal attaching and effacing (A/E) histopathology, which is manifested by a localized degeneration of brush border microvilli and an intimate attachment of bacteria to actin-rich pedestal-like structures formed on the apical membrane directly beneath adherent bacteria [3]. The A/E lesion is due to the activity of a type III secretion system (T3SS) mainly encoded by the 35–45 kb locus of enterocyte effacement pathogenicity island (hereafter named LEE), which is conserved in some EHEC isolates and other A/E pathogens such as enteropathogenic *Escherichia coli* (EPEC), atypical EPEC, rabbit EPEC, *Escherichia albertii* and *Citrobacter rodentium*[4-7]. The LEE pathogenicity island comprises at least 41 genes that mainly are located in five major operons (*LEE15*). The LEE encodes a TTSS, translocator proteins, secreted effectors, regulators, an intimin (adhesin) and a translocated intimin receptor. The LEE-encoded regulators Ler, Mpc, GrlR and GrlA are required for proper transcriptional regulation of both LEE- and non-LEE-encoded virulence genes in response to environmental cues [8-12].

The LEE was acquired by horizontal gene transfer [13] and is regulated by both generic *E. coli*- and pathogen-specific transcription factors. Consequently, the regulation of the LEE reflects characteristics of such genetic elements (For review see [11,14]). Silencing of xenogeneic DNA in bacterial pathogens under conditions unfavorable for infection is important to ensure bacterial fitness [15]. H-NS, which is an abundant pleiotropic negative modulator of genes involved in environmental adaptation and virulence [16-20], is a major silencing factor of horizontally acquired genes [21,22]. H-NS silences genes in the H-NS regulon by various mechanisms. Binding of H-NS to regulatory regions of these genes prevents RNA polymerase from accessing and escaping from promoter DNA, which represents two different mechanisms used by H-NS to silence gene expression (see [23-25] and references therein). H-NS is also a major transcriptional modulator of the LEE pathogenicity island, where it negatively affects the expression of *LEE1-5*, *map* and *grlRA*[26-31]. Further, H-NS binds to regulatory sequences upstream of virulence-associated genes located outside of the LEE including those encoding the long polar fimbriae (*lpf*) required for intestine cell adherence and enterohemolysin (*ehx*) [32,33].

The expression of EHEC virulence genes including those encoded by the LEE is derepressed from the H-NS-mediated transcriptional silencing under physiological conditions that EHEC encounters during infection. Also, LEE expression is growth phase-dependent with maximum expression in early stationary phase [34]. H-NS-mediated silencing of transcription is overcome by the action of DNA-binding H-NS paralogues such as the *LEE1*-encoded global transcriptional regulator Ler (For review see [35]). Ler promotes the expression of many H-NS-repressed virulence genes including those of *LEE1-5, grlRA* and non-LEE-encoded virulence genes such as *lpf* and the virulence plasmid pO157-encoded mucinase *stcE*[26,28,31,36-39]. Thus, Ler antagonizes H-NS in the regulation of many virulence genes, which belong to both the H-NS and Ler (H-NS/Ler) regulons.

The *E. coli* stringent starvation protein A (SspA) is a RNA polymerase-associated protein [40] that is required for transcriptional activation of bacteriophage P1 late genes and is important for survival of *E. coli* K-12 during nutrient depletion and prolonged stationary phase [41-43]. Importantly, SspA down-regulates the cellular H-NS level during stationary phase, and thereby derepress the H-NS regulon including genes for stationary phase induced acid tolerance in *E. coli* K-12 [44]. A conserved surface-exposed pocket of SspA is important for its activity as a triple alanine substitution P84A/H85A/P86A in surface pocket residues abolishes SspA activity [45]. SspA is highly conserved among Gram-negative pathogens [44], which suggests a role of SspA in bacterial pathogenesis. Indeed, SspA orthologs affect the virulence of *Yersinia enterocolitica, Neisseria gonorrhoeae, Vibrio cholerae, Francisella tularensis* and *Francisella novicida*[46-51]. Since *E. coli* K-12 SspA is conserved in EHEC where H-NS negatively modulates virulence gene expression, we asked the question of whether SspA-mediated regulation of H-NS affects EHEC virulence gene expression. Here we study the effect of SspA on the expression of LEE- and non-LEE-encoded virulence genes and its effect on H-NS accumulation in EHEC. Our results show that in an *sspA* mutant elevated levels of H-NS repress the expression of virulence genes encoding the T3SS system rendering the cells incapable of forming A/E lesions. Thus, our data indicate that SspA positively regulates stationary phase-induced expression of H-NS-controlled virulence genes in EHEC by restricting the H-NS level.

## Results and discussion

### SspA positively affects transcription of EHEC virulence genes

**Figure 1:**
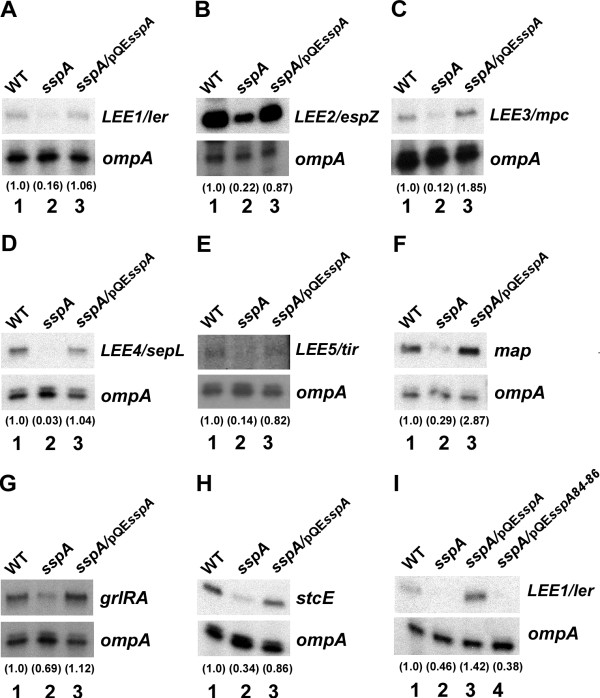
**SspA positively affects LEE expression in stationary phase cells.** Primer extension analyses on total RNA extracted from wild type EHEC EDL933 (lane 1), the *sspA* mutant (lane 2) and the *sspA* mutant complemented with wild type *sspA* (lane 3) or mutant *sspA84-86* (lane 4) as indicated, grown in LB at 37°C to stationary phase (OD_600_ ~ 3.0). The Labeled DNA oligos specific to the transcripts of *LEE1/ler* (**A** and **I**)*, LEE2/espZ* (**B**)*, LEE3/mpc* (**C**)*, LEE4/sepL* (**D**), *LEE5/tir* (**E**), *map* (**F**), *grlRA* (**G**) and *stcE* (**H**) were used. The *ompA* transcripts, detected with a labeled *ompA*-specific DNA oligo, served as internal control for the primer extension reaction. Wild type and mutant SspA were expressed from pQE*sspA* and pQE*sspA84-86* respectively in the absence of induction at similar levels. The transcripts *LEE1-5, map, grlRA, stcE* and the control transcript *ompA* are indicated. The relative transcript levels of target genes normalized to that of *ompA* are indicated by the numbers in parenthesis.

To evaluate the effect of *sspA* on virulence gene expression in EHEC during the stationary phase we constructed an in-frame deletion of *sspA* in the *E. coli* O157:H7 strain EDL933 ATCC 700927 [52] and measured transcription of LEE- (*LEE1-5*, *grlRA* and *map*) and non-LEE-encoded (*stcE* encoded by pO157) genes (Figure 1). Wild type and *sspA* mutant strains were grown in LB medium to stationary phase with similar growth rates (data not shown). Total RNA was isolated and transcript abundance was measured by primer extension analyses using labeled DNA oligos specific to each transcript of interest and *ompA*, which served as internal control for total RNA levels. Results revealed that transcript levels of *LEE1-5*, *grlRA*, *map* and *stcE* were reduced by up to 8-fold in the *sspA* mutant compared to wild type (Figure 1A-H, lanes 1 and 2). The expression of these genes was restored when the *sspA* mutant was supplied with wild type *sspA in trans* from pQE*sspA*[43] (Figure 1A-H, lane 3). However, the expression of *ler* and other virulence genes tested (*grlRA, espZ, sepL* and *stcE*) remained repressed when the *sspA* mutant strain was supplied with mutant *sspA* from pQE*sspA84-86*[45], which expresses SspA containing the triple alanine substitution in the surface-exposed pocket (Figure 1I and data not shown). These results indicate that SspA positively affects stationary phase-induced expression of both LEE- and non-LEE-encoded virulence genes in EHEC. Moreover, the mode of action of SspA is likely similar in *E. coli* K-12 and EHEC as the surface-exposed pocket of SspA also is required for SspA to affect the expression of EHEC virulence genes.

### Increased expression of *ler* enhances expression of virulence genes in the *sspA* mutant

**Figure 2:**
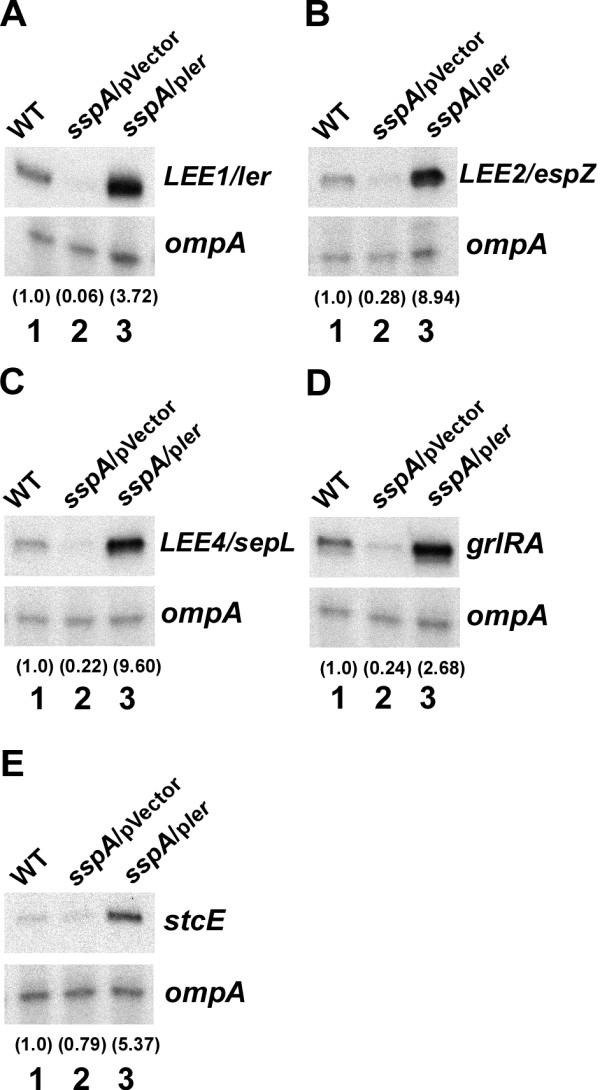
**Increased *ler *expression overcomes repression of LEE in an *sspA *mutant.** The expressions of virulence genes in wild type EHEC EDL933 (lane 1), the *sspA* mutant harboring the empty vector pACYC184 (pVector) (lane 2) and pACYC*ler* (p*ler*) expressing *ler* from its native promoters (lane 3) were determined by primer extension analyses using labeled DNA oligos specific to *LEE1/ler* (**A**)*, LEE2/espZ* (**B**), *LEE4/sepL* (**C**), *grlRA* (**D**) and *stcE* (**E**) along with the *ompA-*specific oligo as a control. Samples were prepared and analyzed as described in the legend of Figure 1. The relative transcript levels of target genes normalized to that of *ompA* are indicated by the numbers in parenthesis.

A decreased expression of *ler* in the *sspA* mutant (Figure 1A) could account for the apparent transcriptional repression of *LEE2-5*, *grlRA*, *map* and *stcE* (Figure 1B-H) because Ler positively controls those genes. Thus, we examined whether supplying *ler in trans* from the plasmid pACYC*ler* would alleviate the expression of Ler-regulated genes in an *sspA* mutant (Figure 2). Our results showed that transcript levels of *LEE1, LEE2, LEE4, grlRA* and *stcE* were all increased in the *sspA* mutant harboring pACYC*ler* and exceeded that in wild type with up to about 9-fold (Figure 2A-E, compare lanes 1 and 3). These results are consistent with the explanation that a reduced expression of *ler* in the *sspA* mutant leads to an insufficient amount of Ler to antagonize H-NS-mediated repression of those virulence genes.

### SspA activates virulence gene expression by reducing the H-NS level

**Figure 3:**
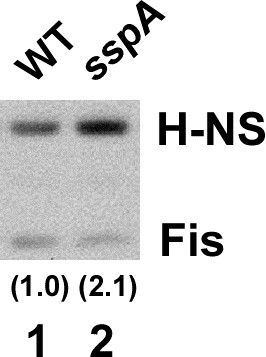
**SspA negatively affects H-NS levels in EHEC.** The levels of H-NS were determined in wild type (lane 1) and *sspA* mutant (lane 2) derivatives of EHEC EDL933 grown to stationary phase cells by western blot. Equal amounts of total protein were resolved on a 10% Bis-Tris SDS-PAGE gel and transferred to a nitrocellulose membrane. Levels of H-NS and Fis were detected using polyclonal antibodies against the respective proteins. Fis served as an internal control for total protein levels. The relative amounts of H-NS normalized to that of Fis are indicated by the numbers in parenthesis.

Reduced virulence gene expression during the stationary phase could also be due to an increased level of H-NS in the EHEC *sspA* mutant as observed for H-NS-regulated genes in the *E. coli* K-12 *sspA* mutant [44]. We measured the levels of H-NS in stationary phase cells of wild type and *sspA* mutant EHEC strains by western analysis (Figure 3). Indeed, the H-NS level was two-fold higher in the *sspA* mutant than in the wild type, whereas the level of Fis as a control was not increased in the mutant compared to wild type. These results indicate that SspA activates the expression of EHEC virulence genes by decreasing accumulation of H-NS. Notably, such relative small change in H-NS levels was previously demonstrated to drastically affect the expression of the H-NS regulon involved in stationary phase-induced acid tolerance of *E. coli* K-12 [44].

**Figure 4:**
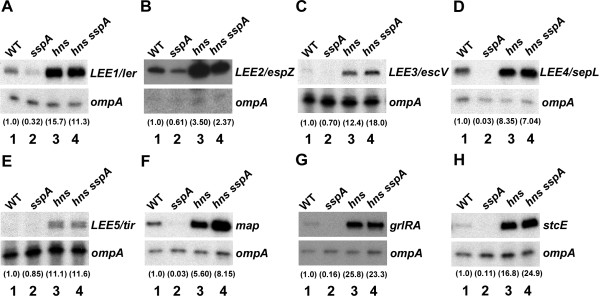
**SspA is upstream of H-NS in the regulatory network of virulence gene expression in EHEC.** The expression of virulence genes in wild type EHEC EDL933 (lane 1), *sspA* (lane 2)*, hns* (lane 3) and *hns sspA* (lane 4) mutant derivatives was determined by primer extension analyses using labeled DNA oligos specific to the transcripts of *LEE1/ler* (**A**)*, LEE2/espZ* (**B**)*, LEE3/mpc* (**C**)*, LEE4/sepL* (**D**), *LEE5/tir* (**E**), *map* (**F**), *grlRA* (**G**) and *stcE* (**H**). In each reaction, the *ompA* transcript served as an internal control. Samples were prepared and analyzed as described in the legend of Figure 1. The relative transcript levels of target genes normalized to that of *ompA* are indicated by the numbers in parenthesis.

Genetic analysis further indicated that *hns* mainly is epistatic to *sspA* in regulating H-NS-repressed virulence genes in EHEC (Figure 4). We deleted *hns* in EHEC wild type and *sspA* mutant strains as described in *Methods*. The EHEC *hns* mutant derivatives had a mucoid phenotype and a longer generation time (g) than wild type (*g*_WT_ ~ 27, *g*_*hns*_ ~ 36 min and *g*_*hns,sspA*_ ~ 45 min). Therefore, at least two independent clones of each *hns* mutant derivative were used in each experiment to ensure reproducible results. The expression of *LEE1-5*, *grlRA*, *map* and *stcE* was between 4 and 26-fold higher in an isogenic *hns* null mutant than in wild type (Figure 4A-H, compare lane 3 with 1), which is consistent with the fact that there is enough H-NS in stationary phase wild type cells (Figure 3) to partially repress those virulence genes. Although the effect of *hns* on cell growth will be complex, an uncontrolled expression of the LEE genes and the T3SS is likely to be detrimental to the fitness of the cell [15]. Moreover, the expression level of EHEC virulence genes in the *hns sspA* double mutant was within the range of the level observed for the *hns* single mutant (Figure 4A-H, compare lane 4 with 3). Thus, our data strongly indicate that SspA is located upstream of H-NS in the regulatory cascade controlling the virulence gene expression in EHEC. However, SspA might also directly activate virulence gene expression in addition to controlling H-NS levels.

### SspA is required for cell adherence and A/E lesion formation

**Figure 5:**
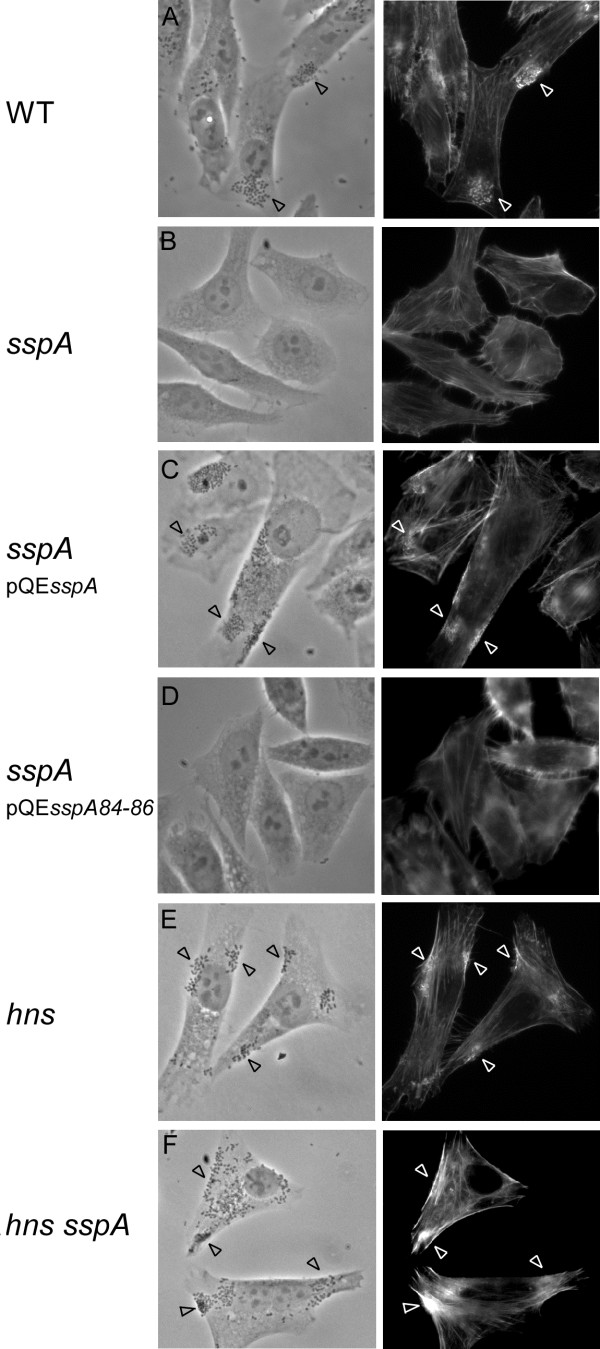
**SspA is required for cell adherence and A/E lesion formation.** HEp-2 cells were infected by wild type EHEC EDL933 (**A**) and its mutant derivatives of *sspA* (**B**), *sspA* pQE*sspA* (**C**), *sspA* pQE*sspA84-86* (**D**), *hns* (**E**) and *hns sspA* (**F**). Bacterial adherence was examined by phase-contrast images (left panels) and the actin cytoskeleton of infected HEp-2 cells by fluorescent microscopic images (right panels). Representative images are shown. Black and white arrowheads indicate bacteria and A/E lesions, respectively.

Since the expression of LEE-encoded genes involved in A/E lesion formation was decreased in a *sspA* mutant and increased in a *hns sspA* double mutant (Figures 1 and 4), we predicted that SspA affects lesion formation in a H-NS-dependent manner. To address this, we infected HEp-2 cells with wild type, *sspA*, *hns* and *hns sspA* mutant derivatives of EDL933, and determined the ability of these strains to form A/E lesions *in vitro*. To this end we used the qualitative fluorescent actin staining (FAS) assay [53], where actin filaments are stained with FITC-phalloidin to detect A/E lesions that are visualized as condensed actin directly beneath adherent bacteria. Whereas infection with wild type EHEC was associated with the appearance of microcolonies of adherent bacteria and A/E lesion formation on 70% of the HEp-2 cells (Figure 5A), the *sspA* mutant was unable to adhere and form A/E lesions (Figure 5B) as determined from examination of more than 50 HEp-2 cells. The A/E lesion phenotype of the *sspA* mutant was restored when complementing with *sspA in trans* from pQE*sspA* (Figure 5C), whereas mutant *sspA* supplied from pQE*sspA84-86* (Figure 5D) did not complement pedestal formation of the *sspA* mutant, verifying that the surface-exposed pocket is functionally important for SspA to affect virulence of EHEC. Consistent with the finding that SspA regulates LEE expression through H-NS, the *sspA* mutant restored the ability to form A/E lesions in the absence of *hns* in the *hns sspA* background as in the *hns* single mutant (Figure 5E-F). However, the *hns sspA* double mutant seemed to form A/E lesions to a higher degree than the *hns* single mutant, which indicates that SspA also affects the expression of virulence genes involved in A/E lesion formation independently of the H-NS-mediated regulation. Moreover, the finding that the cell adherence ability of the *sspA* mutant was restored when deleting *hns* indicates that a factor negatively regulated by H-NS is required for the adherence of EHEC to epithelial cells. The long polar fimbria, LpfA, which is part of the H-NS/Ler regulon and is required for cell adherence of EHEC [32,54,55], might represent such a factor. Altogether, the cell adherence and A/E lesion phenotypes of the *sspA* mutant are consistent with the finding that SspA positively regulates the expression of genes encoding the T3SS including those of the LEE by negatively affecting H-NS levels.

The correlation between the effects of *sspA* on the transcription of H-NS/Ler-regulated virulence genes and on A/E lesion formation upon infection of HEp-2 cells supports the conclusion that SspA upregulates the expression of LEE and other virulence genes by reducing the accumulation of H-NS in the cell. A reduced cellular H-NS level mediated by SspA will derepress the H-NS regulon and thereby allow the expression of transcriptional activators such as Ler and GrlA. These two activators then form a positive transcriptional regulatory loop partially by preventing H-NS-mediated repression [28]. Accumulation of Ler will in turn antagonize H-NS function and with that enhance the expression of virulence genes controlled by Ler [26]. At present, the molecular mechanism behind SspA-mediated regulation of the H-NS level during stationary phase and in infection to facilitate virulence gene expression in EHEC is unknown. Also, it remains to be determined whether SspA directly affects transcription of virulence genes as is the case for SspA in *Francisella tularensis*, where SspA along with two other transcription factors and ppGpp activates transcription to link the nutritional status to virulence gene expression [56,57].

We observed that SspA positively affects additional H-NS-controlled virulence traits of EHEC such as stationary phase-induced acid tolerance (data not shown), which enables survival of the pathogen during passage through the low pH environment of the human gastrointestinal tract, and thereby contributes to a low infectious dose [58,59]. Also, *sspA* positively affects EHEC motility (data not shown), which could influence virulence as motility enables the pathogen to penetrate the intestinal mucus layer during colonization of host cells. This further supports an important role of *sspA* in EHEC virulence. Further experiments studying wild type and *sspA* mutant derivatives of the A/E pathogen *Citrobacter rodentium* in a mouse model could help determine whether *sspA* is required for virulence *in vivo*.

## Conclusions

We established an important role of SspA in the regulation of LEE- and non-LEE-encoded virulence factors of a T3SS, which is important for A/E lesion formation by EHEC. SspA downregulates H-NS levels allowing the expression of EHEC virulence genes, which are part of the H-NS/Ler regulon. Virulence genes in many bacteria are horizontally acquired genetic elements and subject to repression by H-NS. Thus, our study indicates that SspA potentially plays an important role in the pathogenicity of many bacterial pathogens in general.

## Methods

### Standard procedures

Standard DNA techniques, agar plates and liquid media were used as described [60]. Restriction endonucleases, T4 DNA polynucleotide kinase- and ligase (New England Biolabs) and the Expand High Fidelity PCR System (Roche Applied Sciences) were used according to manufacturer’s instructions. DNA sequencing was performed by the National Cancer Institute DNA Sequencing MiniCore facility. Bacteria were grown at 37°C in LB or DMEM (Invitrogen #11885) media supplemented with ampicillin (100μg/ml), chloramphenicol (25 μg/ml) or kanamycin (25 μg/ml) as needed. HEp-2 cells (ATTC # CCL-23) were cultured in DMEM supplemented with 10% fetal bovine serum (FBS), 100 U/ml penicillin and 100 μg/ml streptomycin at 37°C in 5% CO_2_.

### Strain and plasmid constructions

Oligonucleotides used in this study are listed in Table 1. Gene deletions were constructed in EHEC O157:H7 EDL933 strain ATCC 700927 (Perna et al. 2001) by Lambda Red-mediated recombination using linear DNA fragments as described [61]. An in-frame deletion of *sspA* was created as previously described [44] resulting in strain DJ6010 (ATCC 700927 Δ*sspA*). The DNA fragment used for making the *sspA* deletion was amplified by PCR from pKD13 with primers PKD13sspAUS2 and PKD13sspADS. An *hns* deletion mutant derivative of strain ATCC 700927 was made by inserting a chloramphenicol resistance-encoding *cat* cassette, which was PCR amplified from pKD3 [61] using primers Δhns92-1 and Δhns92-2, 276 nt from the *hns* translation initiation codon (strain DJ6011). An *sspA hns* double mutant (DJ6012) was constructed by introducing the Δ*hns::cat* deletion into strain DJ6010. All gene deletion constructs were verified by PCR amplification using primer sets sspABUS/sspABDS and hnsUS2/hnsDS2. In addition, Western blot analysis using polyclonal antibodies specific to the respective proteins confirmed the *sspA* and *hns* mutant strains. Plasmid pACYC*ler* (pDJ610) contains a ~ 800 bp DNA fragment encoding *ler* expressed from its two native promoters cloned into the *Hind*III/*Bam*HI sites of pACYC184. The DNA fragment was PCR amplified from EDL933 genomic DNA using oligos lerUS2/lerDS2.

**Table 1:** Oligonucleotide primers used in this study

**Application**	**Name**	**Oligonucleotide sequence (5’ to 3’)**
*Strain construction*	PKD13sspAUS2	ACTATCATCCAATTTTCTGCCCAAATGTCGGGTATTGCTCAGGAGGTTTCTTTCATGATGTCCGGGGATCCGTCGACCTGC
	PKD13sspADS	AGATTAACTCCGGCCCAGACGCATTTCACGTTCTGCTTCAGTGTAGGCTGGAGCTGCTTCGA
	sspABUS	GATTGGATCCGGCTTCGTTACTCGTGACGCT
	sspABDS	GATTGAAGCTTACTTCACAACGCGTAATGC
	hnsΔ92-1	GAGCTGCTGAATAGCCTTGCCGCCGTTAAATCTGGCACCAAAGCTAAACGTGCTTGTGTAGGCTGGAGCTGCTTCG
	hnsΔ92-2	GGTTTTAGTTTCGCCGTTTTCGTCAACGTAGCTATATTTTGCCGGACGCTTATGAATATCCTCCTTAGTTC
	hnsUS2	CCATGGAATTCACCATGAGCGAAGCACTTAAAATTCTGAACA
	hnsDS2	CCAGGTCTGCAGTTATTGCTTGATCAGGAAATCG
*Plasmid construction*	lerUS2	GATTGGATCCGCTGCGACTGCGTTCGCTTGCT
	lerDS2	GATTGAAGCTTCCAGCTCAGTTATCGTTAT
*Primer extension*	lerPE	GCTTCCTGCTGTAGA
	LEE2PE	GCTGCTTCCATTGATCTTTCTCC
	LEE3PE	CAATTTCAACACGGTTATC
	LEE4PE	CAGATGCGGGGTTTTGATTAAATTC
	LEE5PE	CATTGGGATTATGACCAAGA
	mapPE	GGACTAAACATGCTATAAACC
	grlRPE	CCTTCCCCACAGGAGTCTTC
	stcEPE	GGCAAGGATCGTACATGA
	ompAPE	ACGAGATAACACGGTTAAATCC

### RNA isolation

DMEM is known to enhance the expression of the T3SS, which was detrimental to growth of the *hns* mutant EHEC derivatives (data not shown) that already exhibit increased T3SS expression in the absence of H-NS-mediated repression. Therefore, virulence gene expression was monitored in cells grown in LB, where a mid-level expression of the T3SS occurs. Overnight cultures of wild type and mutant derivatives of EDL933 ATCC 700927 were diluted 1:1000 in LB, supplemented with antibiotics if necessary, and grown aerobically at 37°C to an optical density at 600 nm (OD_600_) of ~ 3.0 (stationary phase). Samples of the cultures corresponding to ~7.5 × 10^9^ cells were collected and RNA was stabilized immediately by addition of RNAprotect bacteria reagent according to manufacturer’s protocol (QIAGEN). Total RNA was purified using MasterPure™ total RNA purification kit as recommended by the manufacturer (Epicentre). Contaminating DNA in the RNA preparations was removed by DNaseI treatment. Isolated RNA was quantified based on measurements of absorption at 260 nm. The quality of RNA was evaluated by determining the ratio of absorption at 260 nm and 280 nm, which was within the preferred range of 1.7 to 2.1, and by agarose gel electrophoresis.

### Primer extension analysis

Primer extension reactions were carried out on 8 μg of total RNA using the AMV reverse transcriptase primer extension system according to manufacturer’s instructions (Promega). The ^32^P-labeled DNA oligos (1 pmol) were used to detect the transcripts of interest: lerPE (*LEE1/ler*), LEE2PE (*LEE2/espZ*), LEE3PE (*LEE3/mpc*), LEE4PE (*LEE4/sepL*), LEE5PE (*LEE5/tir*), mapPE (*map*), grlRPE (*grlRA*) and stcEPE (*stcE*). The ^32^P-labeled DNA oligo ompAPE was used as an internal control in each extension reaction to detect the transcripts from *ompA* P1 and P2. The DNA oligos were 5’-end labeled with (γ-^32^P) ATP (GE Healthcare) using T4 polynucleotide kinase (New England Biolabs). The cDNA products were separated on a 6 % denaturating gel along with a labeled φX174 *hin*fI DNA size marker (Promega) and visualized by autoradiography. The lengths of the cDNA transcripts *ler, espZ, mpc, sepL, tir, map*, *grlRA, stcE, ompA* P1, and *ompA* P2 were around 250, 120, 147, 122, 142, 135, 162, 125, 89 and 93 nt, respectively. No DNA product was detected in the absence of RNA. Transcript levels were quantified using ImageJ software [62] and normalized to *ompA* transcript levels. The primer extension experiments were carried out at least twice and similar results were obtained.

### Western analysis

Total protein was prepared from cultures grown in LB at 37°C to OD_600_ ~ 3.0. Samples containing equal amounts of total protein equivalent to 0.03 OD_600_ units of cell culture were prepared and analyzed essentially as previously described [44]. Polyclonal antibodies against H-NS or Fis were used to detect the respective proteins. The western blots were developed using ECL plus reagents (GE Healthcare) and quantified with a FluorChem imaging system (Alpha Innotech). The western analysis was carried out at least twice, and similar results were obtained.

### Assay for the presence of A/E lesions on HEp-2 cells

The ability of EHEC EDL933 (ATCC 700927) wild type and its mutant derivatives to adhere and form A/E lesions on HEp-2 cell monolayers was evaluated using the fluorescent actin staining assay as described [53]. Bacterial cells were grown without aeration for 16–18 h at 37°C in tryptic soy broth that was supplemented with antibiotics if needed. Prior to infection cells were diluted 1:5 in infection medium (DMEM supplemented with 2% FBS and 0.5% mannose) and incubated at 37°C 5% CO_2_ for 2 h. About 2 × 10^6^ bacteria (M.O.I. ~ 10) in 100 μl were added to semi-confluent HEp-2 cell monolayers grown on glass coverslips in a 6-well plate (Multiwell™ Falcon #353046). After infection for 4–5 h, monolayers were fixed with 4% formamide in PBS, washed three times with PBS, permeabilized with 0.1% Triton X-100 in PBS, and then stained with Alexa Fluor 488 phalloidin (Invitrogen). Coverslips were mounted on slides using Prolong Gold antifade reagent (Invitrogen) and the edges of the coverslip were sealed with cytoseal-60 (Richard-Allan Scientific). The samples were visualized using a Zeiss Axiophot II microscope equipped with a 40X objective, epifluorescence filters and a 1.25 optovar (Carl Zeiss MicroImaging Inc.). Images were captured with a charge-coupled device camera (Micromax) using IPL lab software. For each bacterial strain the assay was carried out independently at least three times and at least 50 HEp-2 cells were visually examined.

## Competing interests

The authors declare that they have no competing interests.

## Authors’ contributions

Experiments were designed by AH and DJ. Experiments were performed by AH. The manuscript was written by AH and DJ. Both authors have read and approved the final manuscript.
